# Canine Ticks, Tick-Borne Pathogens and Associated Risk Factors in Nigeria

**DOI:** 10.3390/pathogens14121271

**Published:** 2025-12-11

**Authors:** Ternenge Thaddaeus Apaa, Philip Oladele Oke, Felix Kundu Shima, Gberindyer Aondover Fidelis, Stephen Dunham, Rachael Tarlinton

**Affiliations:** 1 School of Veterinary Medicine and Science, University of Nottingham, Sutton Bonington Campus, Loughborough, Leicestershire LE12 5RD, UKrachael.tarlinton@nottingham.ac.uk (R.T.); 2 College of Veterinary Medicine, Joseph Sarwuan Tarkaa University, Makurdi P.M.B 2373, Benue State, Nigeria; 3 Virology Department, Animal and Plant Health Agency, Woodham Lane, Addlestone KT15 3NB, UK; 4 Department of Animal Science, School of Agriculture, Policy and Development, University of Reading, Whiteknights, Reading RG6 6EU, UK; 5 Department of Veterinary Medicine, University of Ibadan, Ibadan P.M.B 001, Oyo State, Nigeria; 6 Faculty of Veterinary Medicine, Department of Veterinary Medicine, University of Benin, Ugbowo, Benin City P.M.B 1154, Edo State, Nigeria

**Keywords:** canine ticks, tick-borne pathogens, risk factors, Nigeria

## Abstract

Tick-borne pathogens (TBPs) pose a significant threat to canine health in Nigeria. Despite this, there is little data on the molecular identification of ticks and TBPs of dogs in Nigeria. This study assessed the prevalence of ticks and TBPs in Nigerian dogs, along with associated risk factors. A total of 259 dogs were enrolled in the study, from which 112 adult ticks were collected. Of these, 40 were characterized by molecular barcoding confirming *Rhipicephalus sanguineus* (*R. sanguineus*, 35/40) and *Haemphysalis leachi* (*H. leachi*, 5/40) infestations. Nucleotide sequences showed high percentage similarity to *R. sanguineus* tropical lineage and *H. leachi* sequences from Chad. Point-of-care (POC) testing of 259 dogs detected antibodies to TBPs in 40.9% of blood samples, with *Ehrlichia* (29.7%), *Anaplasma* (10.8%), and *Dirofilaria* (0.4%) species identified. PCR assays revealed a prevalence of 58.7% for TBPs, including *Ehrlichia* (40.5%) and *Babesia* (17.4%), with 7.3% co-infected. Risk factor analysis showed that adult dogs and those infested with ticks had a higher likelihood of TBP seropositivity. Exotic breeds and dogs examined during the rainy season were more likely to test positive for TBPs via PCR. Overall, this study demonstrates the high prevalence of diverse TBPs in Nigerian dogs and suggests that dog breed may play a role in susceptibility to diseases.

## 1. Introduction

Canine ticks transmit a range of infectious pathogens and pose a significant threat to both animal and human health globally [1,2,3]. Over 877 species belong to two major families: *Ixodidae* (hard ticks) and *Argasidae* (soft ticks), which are distributed globally [4]. In Nigeria, like in other tropical regions, ticks are pervasive and diverse. While *Argasidae* primarily infest birds, certain members can transmit pathogens to livestock and humans, whereas *Ixodidae* remain the primary vectors for disease transmission to livestock, companion animals, and humans [5]. Ticks infesting domestic dogs can induce various clinical manifestations, including skin irritation, wounds, paralysis, anemia, and even death [6,7].

Canine tick-borne pathogens (TBPs) encompass a spectrum of viruses, bacteria, and parasites, including protozoa and helminths, capable of infecting canines. Among the most prevalent TBPs are *Ehrlichia*, *Anaplasma*, *Babesia*, *Hepatozoon*, *Borrelia*, *Bartonella*, and *Rickettsia.* However, the *Dirofilaria* species assessed in this study is classified as a mosquito-borne pathogen of dogs [8].

Infections with multiple species of TBPs are frequent in dogs and often manifest with nonspecific clinical signs [9,10]. Moreover, the zoonotic potential of many canine tick-borne diseases (TBDs) highlights the public health significance of tick control [11]. Consequently, effective control of ticks is crucial for safeguarding both animal and human populations.

Several factors influence tick life cycles and transmission of tick-borne pathogens including climate, host availability and susceptibility to TBPs infection, vegetation cover, and predation. Climate change, particularly in regions like Nigeria, can alter tick distribution and abundance, potentially increasing the risk of TBDs [12,13].

Tick control measures in Nigeria involve mechanical removal and acaricide application. However, the efficacy of these methods is challenged by acaricide resistance, limited availability of safe and effective products [14], and the lack of effective vaccines for canine TBDs [15,16]. In Nigeria, where ticks pose a considerable threat to animal and human health, understanding the dynamics of tick infestation and implementing effective control strategies are imperative for mitigating the impact of TBDs.

Previous studies in Nigeria focused primarily on tick distribution in livestock using morphological identification methods, with limited information available on canine tick infestation [3,17,18,19]. There is a critical gap in understanding the population distribution of diverse tick species infesting dogs across Nigeria.

Despite the significance of TBPs, reports on their prevalence in Nigeria have been limited, often focusing on one or two pathogens and specific regions within the country [20,21]. In addition, comprehensive information on risk factors associated with TBP infection in dogs using combination of Point-of-Care (POC) and polymerase chain reaction (PCR) diagnostics has not been established.

This study made efforts to address this gap by employing molecular barcoding methods to confirm the identity of tick species infesting Nigerian dogs and detected multiple TBPs through POC and PCR diagnostics. Risk factors associated with dogs infested with ticks and TBPs were also evaluated. By elucidating the prevalence and associated risk factors of TBPs in Nigerian dogs, this research aims to enhance our understanding of canine ticks and TBPs and inform targeted control and prevention strategies.

## 2. Materials and Methods

### 2.1. Ethical Approval, Questionnaire Design/Consent Form and Sample/Demographic Data Collection

This study was approved by the University of Nottingham School of Veterinary Medicine and Science Committee for Animal Research and Ethics (Ethical approval number: 2416180702). Random sample collection was applied following the utilization of the online epidemiological tool server (https://epitools.ausvet.com.au/oneproportion, accessed 29 May 2018) to estimate sample size at 95% confidence level using a mean prevalence of 41.8% [21,22] from Nigeria (Supplementary File S1: Table S1). A structured questionnaire and sample collection form (Supplementary File S2) was used to obtain demographic data on dogs tested for tick-borne pathogens via the POC (IDEXX^®^) test kit and consent from participating clients. Due to low levels of literacy and the plethora of languages among participants, verbal consent was obtained by participating veterinarians. Recorded variables included dog identification and history, vaccination status, and demographic details such as sex, age category, and breed. Physical condition was assessed using body weight and movement status (stray, non-stray, unknown). Tick infestation was categorized as mild (1–5 ticks), moderate (5–10 ticks), or heavy (>10 ticks), with total tick counts and species identified morphologically.

A total of 112 ticks were collected from 259 domestic dogs (June 2018–November 2019) sourced from four locations across three geopolitical zones in Nigeria: Makurdi in Benue State (North central), Zaria in Kaduna (Northwest), and Ibadan in Oyo State (Southwest). The collection process was conducted by trained veterinarians. A minimum of four ticks were collected from each infested dog and preserved in 70% ethanol and subsequently subjected to morphological identification according to established protocols [23]. Further processing involved grinding collected ticks in a porcelain mortar and pestle, followed by placement onto Whatman’s FTA cards and air drying at ambient temperature prior to shipment.

In addition to tick samples, blood specimens (2 mL) were aseptically obtained from all 259 dogs using sterile EDTA tubes. Subsequently, a commercial point-of-care (POC) test, specifically SNAP^®^ 4Dx^®^, was used to test these blood samples for the presence of antibodies against TBPs. All remaining blood samples were placed onto Whatman’s FTA cards, air dried at ambient temperature, and packaged in plastic zip bags containing silica gel desiccants for preservation during shipment.

All samples, point-of-care (POC) results, and demographic data collected were transported to the University of Nottingham for subsequent analysis.

### 2.2. IDEXX^®^ Antigen Kit Testing on Canine Blood

Following sample collection, participating veterinarians conducted SNAP^®^ 4Dx^®^ POC test on blood samples for the detection of antibodies against canine TBPs, adhering to the manufacturer’s instruction (SNAP^®^ 4Dx^®^ test kit by IDEXX Laboratories, Inc., Westbrook, ME, USA). The POC test kit detects antibodies against membrane protein *VIsE* of *Borrelia burgdorferi* (*B. burgdorferi*) (s.l.) and the membrane protein *p30*/*p30-1* of *Ehrlichia canis* (*E. canis*). It also detects antibodies against *p28* outer surface protein derived from other *Ehrlichia* sp. including *E. chaffeensis*, *E. canis*, and *E. ewingii* [24]. Additionally, this kit also detects antibodies against the major surface protein (*p44*/*MSP2*) of *Anaplasma phagocytophilum* (*A. phagocytophilum*)/*Anaplasma platys* (*A. platys*) [25] and *Dirofilaria immitis* (*D. immitis*) antigen [24]. *D. immitis* antigen POC detection kits may cross-react with other nematodes like *Dirofilaria repens* (*D. repens*), *Dipetalonema reconditum*, *Onchocerca* sp., *Spirocerca lupi*, and *Angiostrongylus vasorum*, especially the latter without prior heat pre-treatment of sera [26,27].

### 2.3. DNA Extraction and Polymerase Chain Reaction, Gel Electrophoresis and Sanger Sequencing

DNA extraction from canine tick and blood samples preserved on FTA cards utilized the Macherey Nagel Nucleospin kits, following the manufacturer’s protocols. The protocol for purification of genomic DNA from insects was applied to tick samples, while the protocol for genomic and viral DNA from blood samples was used for blood collected on FTA cards. For both sample types, the pre-lysis step was modified by adding 5 mm stainless steel beater beads (QIAGEN^®^ Ltd., Manchester, UK) to each tube, followed by incubation in a heat block for 1–2 h as recommended. Tubes were vortexed every 15 min during incubation to ensure effective lysis, homogenisation, and improved DNA yield before proceeding with the remaining steps of the manufacturer’s instructions. DNA concentration and quality was assessed using a Nanodrop 8000 spectrophotometer (Thermo Scientific^®^, Waltham, MA, USA).

### 2.4. Hard 16S rRNA Gene PCR

Hard tick generic primers (16S + 1 and 16S − 1) were utilized to amplify a 460 bp segment of the 16S rRNA gene in tick samples [28]. PCRs were conducted in a 50 μL master mix containing 1× standard Taq (Magnesium-free) buffer (BioLabs^®^, Cambridge, MA, USA), 200 μM dNTP (BioLabs^®^), 1.5 mM magnesium chloride, 0.2 μM of each primer, 34.5 μL of RNase-free water (Sigma-Aldrich, Irvine, UK), 1.25 units of taq DNA polymerase (BioLabs^®^), and 5 μL of DNA template. PCR thermal cycling (Bio-Rad, Hercules, CA, USA) conditions consisted of initial denaturation at 94 °C for 3 min, followed by 40 cycles of denaturation at 94 °C for 1 min, annealing at 54 °C for 30 s and extension at 72 °C for 90 s, and one cycle of final extension at 72 °C for 5 min (Supplementary File S1: Table S2). *R. sanguineus* DNA was used as the positive control (courtesy of Dr Lesley Bell-Sakyi, The Tick Cell Biobank research group, University of Liverpool UK).

### 2.5. Babesia, Ehrlichia, Borrelia, and Dirofilaria PCR Detection

Previously published pan piroplasm PCR primer pairs (Piro-A and Piro-B) were used to amplify a 400 bp segment of the *Babesia* 18S rRNA gene in canine blood samples [2,29]. The previously published generic conventional PCR primers, originally designed for detection of *Ehrlichia* and *Anaplasma* species [30], were used for the detection of *Anaplasma* and related species in canine blood samples. Additionally, specific qPCR primers (Ecp30-f and Ecp30-r) targeting an 80 bp region of the *E. canis* p30 gene were used for *E. canis* detection [31]. The presence of *Borrelia* species in canine blood samples was assessed using generic qPCR primers (Bor16S3F and Bor16S3R) to amplify 148 bp of *Borrelia 16S* RNA [32]. Detection of *Dirofilaria* used published filariid-specific qPCR primers targeting a 90 bp fragment of the filarial species 16S rRNA gene [33].

The *Ehrlichia*/*Anaplasma* generic conventional PCR consisted of 50 μL reaction volume including 1× standard Taq (Magnesium-free) buffer (BioLabs^®^), 200 μM dNTP (BioLabs^®^), 1.5 mM magnesium chloride, 0.2 μM of each primer, 34.5 μL of RNase-free water (Sigma-Aldrich, UK), 1.25 units of taq DNA polymerase (BioLabs^®^), and 5 μL of DNA templates. PCR thermal cycling (Bio-Rad) conditions comprised initial denaturation step at 94 °C for 3 min, followed by 40 cycles of denaturation at 94° C for 45 s, annealing at 50 °C for 30 s, extension at 72 °C for 90 s, and a final extension step at 72 °C for 90 s.

All qPCR reactions for *Babesia*, *E. canis*, *Borrelia,* and filarial species were conducted in a 20 μL master mix, comprising 10 μL 2× qPCRBIO SyGreen Mix Lo-ROX (PCR Biosystems, London, UK), 0.4 μM (0.8 μL) of each forward and reverse primer, 4.2 μL of RNase-free water (Sigma-Aldrich, UK), and 5 μL of template DNA. Consistency was ensured with triplicate qPCR reactions per sample. Initial specific pathogen gradient and qPCR optimization for *Babesia*, *Ehrlichia*, *Borrelia*, and *Dirofilaria* species using the Bio-Rad CFX real-time qPCR machine yielded identical annealing temperature (60 °C for 45 s) and thermal cycling conditions for all samples, maintaining adequate qPCR efficiency (≥96%), strong linearity demonstrated by high coefficient of determination (R^2^ ≥ 0.97), and quantification cycle threshold (Cq). Thermal cycling conditions included initial denaturation at 98 °C for 3 min, followed by 40 cycles of 98 °C for 15 s and a combine annealing/extension at 60 °C for 45 s. qPCR analysis was performed using Bio-Rad CFX Maestro 1.1 software. Pathogen DNA controls were kindly donated by various researchers (*Babesia*: Dr Chris Helps; Molecular Diagnostic Unit, Langford Vets Bristol UK and Dr Nicholas Johnson; APHA, UK; *E. canis*: Dr Lesley Bell-Sakyi; The Tick Cell Biobank, University of Liverpool UK; *B. burgdorferi* (s.l.): Dr Daniel Carter; PHE, UK; and *D. immitis*: Dr Silvana Belo; Global Health and Tropical Medicine University of Lisbon, Portugal). Canine GAPDH PCR was used to confirm DNA quality prior to pathogen PCR detection [34]. Further details of PCR primers and thermal cycling conditions used in this study are provided in Supplementary File S1: Table S2.

### 2.6. Agarose Gel Electrophoresis and Sanger Sequencing and Analysis

PCR bands were imaged on Nancy 520 stained 1% agarose TAE gel and electrophoresed at 80 volts for 60 min. Gel images were captured using an Imagequant 300 UV camera (DOTmed.com Inc., New York, NY, USA). Tick, *Babesia*, and *Ehrlichia* PCR products were purified using the Nucleospin^®^ extract II kit (Macherey-Nagel) and subjected to Sanger sequencing (Source BioScience UK) using specific primers corresponding to each target product. The molecular identification of the 40 ticks sequenced was consistent with the initial morphological identification performed during field sampling. Therefore, sequencing the remaining tick sample PCR products was deemed unnecessary, as this would have incurred additional costs without providing further benefit.

Sanger sequence quality assessment, editing, assembly, and consensus sequence nucleotide generation were conducted in Geneious Prime^®^ software 2019 v11.0.3+7 [35]. Confirmation of tick and tick-borne piroplasm nucleotide sequence identity was performed through NCBI online Blastn searches [36]. Sequence identified in NCBI database during Blastn search and relevant published literature on tick [28,37,38,39,40] and *Babesia* sp. taxonomy [2,41] were downloaded for subsequent phylogenetic analysis. For each phylogenetic tree involving ticks (*Rhipicephalus* sp., *Haemaphysalis* sp.) and *Babesia* sp., the downloaded sequences were aligned using MAFFT, assessed, and trimmed to ensure uniform length in Geneious Prime^®^ software 2019 v11.0.3+7. Maximum-likelihood phylograms were then constructed in IQ-TREE v2.0.7 with 1000 bootstrap approximations, utilizing the best nucleotide substitution model automatically selected [42]. This included the Tamura-3-parameters-T92 model for ticks [43] and the Jukes–Cantor for *Babesia* sp. [44], selected based on the lowest Bayesian Information Criterion (BIC) and evaluation of tree nodes via implementation of UFBoot2.

### 2.7. Descriptive Statistics and Multivariate Modelling

Descriptive statistics, univariate and multivariate modelling to evaluate risk factors associated with TBPs prevalence in canine blood using POC and PCR tests, and tick infestation were performed using IBM SPSS statistical software v.26.0. The multivariate modelling aimed to identify predictors of TBPs infection in dogs, excluding *Dirofilaria* detection (a mosquito-borne infection in dogs). A top-down (backward) multivariate modelling strategy was employed, initially assessing significant exposure variables, followed by backward stepwise elimination to retain significant predictors identified as co-founders (*p* < 0.05 and/or adjusted regression coefficient (R2) of >10%). Cohen’s kappa index (κ) was estimated at 95% confidence interval using IBM SPSS statistical software v.26.0 to determine agreement between the POC and PCR-based diagnostic tests. Kappa values were interpreted as follows: poor (κ < 0); light (κ = 0–0.20); fair (κ = 0.21–0.40); moderate (κ = 0.41–0.60); substantial (κ = 0.61–0.80); and excellent (κ = 0.81–1.00) [45].

## 3. Results

### 3.1. Canine Tick Morphology and Molecular Barcoding

**Figure 1:**
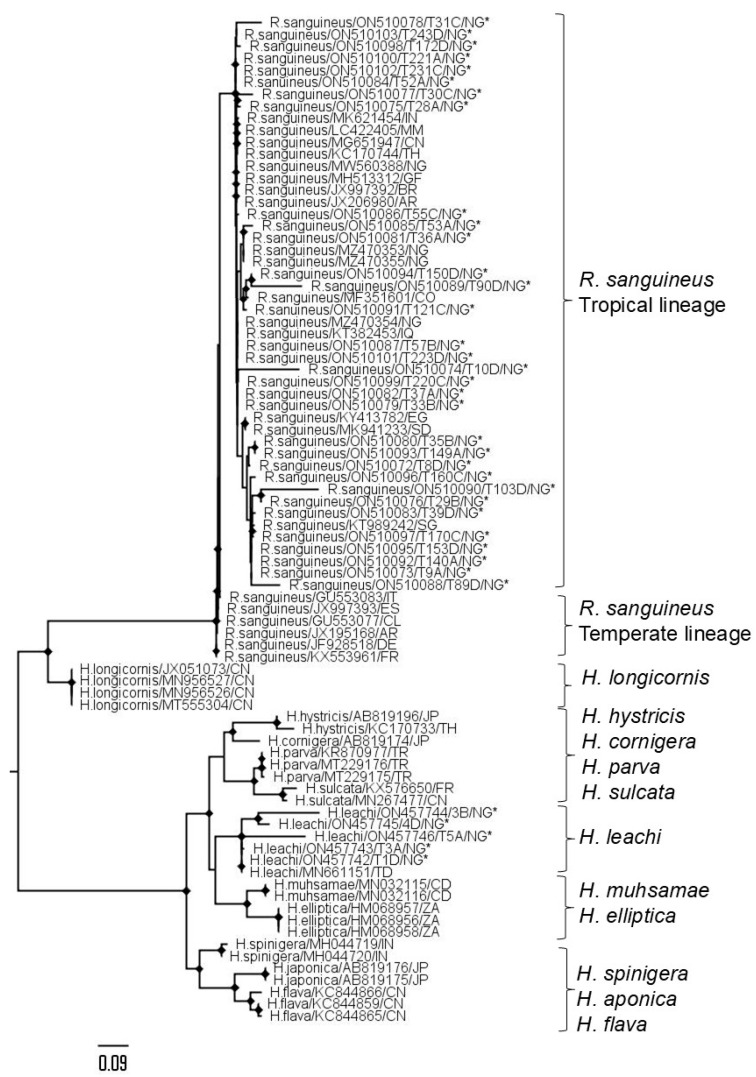
Maximum-likelihood phylogenetic tree of partial tick 16S nucleotide sequences. Named by species, GenBank accession number, and country of origin. Bootstrap support values ≥70% are marked with diamonds on nodes. Sequences from this study are marked with an asterisk. All sequences are labelled with the tick genus, species, NCBI accession number, and two-letter country code.

A total of 112 adult ticks infesting dogs were examined in this study (Supplementary File S1: Table S3). Of these samples, 40 were subjected to Sanger sequencing, yielding a 400 bp nucleotide sequence (Supplementary File S1: Figure S1). Analysis revealed the presence of *R. sanguineus* (35/40) and *H. leach* (5/40) (Figure 1; Supplementary File S1: Table S3). Following NCBI online Blastn searches, the *R. sanguineus* sequences showed high similarity to the tropical lineage of *R. sanguineus*, while *H. leach* sequences exhibited high similarity to the *H. leachi* type sequence (MN661151) from Chad, Africa, by Thompson, Dominguez [40]. Molecular barcoding/Sanger sequencing results were consistent with morphological identification conducted during field sampling.

### 3.2. Characteristics of the Canine Population Assessed for TBPs Using POC and PCR

A total of 259 dogs were examined (June 2018–November 2019), categorized by weight: ≤20 kg (*n* = 172) and ≥21 kg (*n* = 87), across multiple locations including Makurdi (*n* = 223), Zaria (*n* = 14), Abuja (*n* = 12) and Ibadan (*n* = 10), during both the rainy (*n* = 181; April–October) and dry season (*n* = 78; November–March). Dogs were further classified by age: <6 months (*n* = 63) and >6 months (*n* = 196), by gender: male (*n* = 154) and female (*n* = 105), and by breed: exotic (*n* = 179) and indigenous (*n* = 80). Information regarding the presence or absence of tick infestation was recorded for each dog, with 153 dogs having history of tick infestation and 106 dogs without. However, tick samples were only available from 110 out of the 153 dogs with history of tick infestation (Supplementary File S1: Table S4). Additionally, two *H. leachi* samples were obtained from one dog (T3A and T3B), bringing the total number of tick samples assessed to 112 (Supplementary File S1: Table S3, Figure 1).

Of the 259 samples assessed, 40.9% (106/259) tested positive for antibodies to TBPs via the POC test kit, including *Ehrlichia* (29.7%: 77/259), *Anaplasma* (10.8%: 28/259), and *Dirofilaria* (0.4%: 1/259) species. None of the samples tested positive for *B. burgdorferi* (s.l.). Dual seropositivity were observed to *Ehrlichia* and *Anaplasma* species (7.7%: 20/259) and both *Dirofilaria* and *Ehrlichia* (0.4%: 1/259) (Table 1A).

### 3.3. Prevalence of TBPs Based on PCR

**Figure 2:**
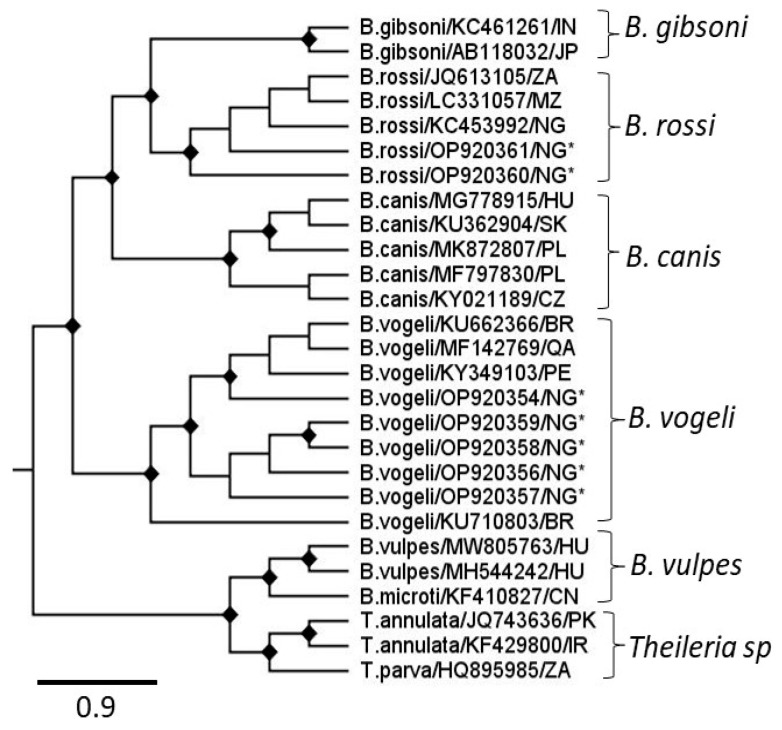
A maximum-likelihood phylogenetic tree constructed with 1000 bootstrap approximations, rooted at midpoint using piroplasm partial 18S nucleotide sequences downloaded from NCBI GenBank and this study (marked with asterisks). Node labels indicate bootstrap support values ≥70%. All sequences are labelled with the tick genus, species, NCBI accession number, and two-letter country code.

Further detection was aimed at validating the prevalence of initial findings observed from the POC test kits. An additional pathogen (*Babesia* sp.), present in Nigeria but not covered by the POC test kit, was also included to produce a more comprehensive analysis of canine TBPs in Nigeria. Overall, 58.7% (152/259) of the canine blood samples assessed were positive for at least one canine TBP following PCR, including *Ehrlichia* (40.5%: 105/259) and *Babesia* (17.4%: 45/259), with 7.3% (19/259) exhibiting co-infection with both *Ehrlichia* and *Babesia* species. To ensure consistency, samples identified to be positive via the piroplasm qPCR yielding PCR products ≥ 400 bp segment were confirmed through Sanger sequencing. Some of the *Babesia*-positive samples could not be identified at the species level due to inadequate Sanger sequences quality. For *Babesia* species, quality nucleotide sequences were generated from 8/45 *Babesia* 18S gene qPCR products including *Babesia vogeli* (*B. vogeli*, 6/8) and *Babesia rossi* (*B. rossi*, 2/8) (Figure 2). Phylogenetic analysis confirmed the presence of *B. vogeli* and *B. rossi* in Nigerian dogs in this study. Of the samples tested, 40.5% (105/259) were positive for *E. canis* DNA via the *E. canis* p30 gene qPCR. Additionally, 2.7% (7/259) of canine blood samples tested positive for *Ehrlichia* using the generic PCR primers (GE2F/EHRL3), with only 0.8% (2/259) detection of *Anaplasma* sp. following Sanger sequencing and NCBI Blastn searches. All samples positive (*n* = 7) via the generic *Ehrlichia* PCR were also positive for *E. canis* using qPCR for the *E. canis* p30 gene. The *Erlichia* sequences were too short for phylogenetic analysis.

None of the blood samples tested positive for *B. burgdorferi* using the generic qPCR assay for *Borrelia* sp., nor for *Dirofilaria* sp. using filaroid-specific qPCR assays (Table 1). All samples included in this study tested positive for canine GAPDH, confirming adequate DNA for PCR.

### 3.4. Risk Factors Associated with Canine TBPs Based on PCR Detection

Multivariate modelling was conducted on six exposure variables, focusing solely on *Ehrlichia* and *Anaplasma* sp., using POC positive results. A dog was considered positive if seropositive for either pathogen, as other pathogen results were negative or recorded ≤1 positive samples. Univariate and multivariate logistic regression modelling for tick-borne pathogens revealed that adult and young dogs exhibited a higher likelihood of seropositivity to TBPs compared to puppies (95% CI 0.15–0.64; *p* = 0.002) (Table 2A; Supplementary File S1: Table S5). Increased risk of TBPs’ seropositivity was correlated with canine tick infestation, with male dogs demonstrating higher likelihood of tick infestation (Table 2B), Supplementary File S1: Table S4).

Logistic modelling assessed risk factors associated with dogs testing positive for one or both *Babesia* sp. and *Ehrlichia* sp. PCR assays (as very few dogs tested positive for *Anaplasma* and these dogs were also positive for *Erlichia*). Non-indigenous dogs were more likely to test positive for *Babesia* and *Ehrlichia* DNA compared to Nigerian indigenous breeds (95% CI 0.28–0.81; *p* = 0.007). Dogs examined during the dry season were less likely to test positive for *Babesia* and *Ehrlichia* DNA via qPCR compared to those assessed during the rainy season (95% CI 0.29–0.87; *p* = 0.014) (Table 3; Supplementary File S1: Table S6). Cohen’s Kappa index values (95% confidence interval) estimated based on POC and qPCR detection of canine blood samples showed poor agreement (κ < 0) between the two testing methods utilized in this study (Table 4).

## 4. Discussion

### 4.1. Tick Species Infesting Nigerian Dogs

The findings from this study shed light on the prevalence and genetic diversity of ticks infesting domestic dogs in Nigeria. *R. sanguineus* (the brown dog tick) was identified as the most abundant species, comprising 95.5% of the total tick population examined, followed by *H. leachi* at 4.5%. Both morphological and molecular identification methods yielded consistent results, indicating the reliability of morphological techniques for identification of these tick species. These findings align with previous studies reporting *R. sanguineus* [3,17,18,46,47,48,49] and *H. leachi* [17,50] as the predominant tick species infesting Nigerian domestic animals, including dogs.

Phylogenetic analysis revealed the close relationship of *R. sanguineus* (s.l.) identified in this study to tropical lineage of *R. sanguineus* (s.l.) from Asia (Singapore, Iraq), South America (Columbia), and Africa (Nigeria, Egypt, Sudan), consistent with *R. sanguineus* (s.l.) species reported [39]. They were distant from temperate lineages in Europe (Germany, France, Spain), and South America (Argentina, Chile). Geographical similarity between Asia, South America, and Sub-Saharan Africa characterized by elevated ambient temperatures is likely to contribute to the distribution of the *R. sanguineus* (s.l.) “tropical lineage” across these regions.

*H. leachi* reported infesting Nigerian dogs were closely related to those from Chad (MN661151) [40] and Nigeria. *H. leachi* prevalence and its role in *B. rossi* transmission has been reported in [50].

### 4.2. Detection of Babesia, Ehrlichia sp. and Anaplasma sp. in Nigerian Dogs Using POC and PCR Tests

The POC testing kit utilized is designed for use in North America and does not include *Babesia* sp. pathogens, which had a high detection rate using PCR in this study, both in single and dual infections with *Erlichia* sp. Consistent with other Nigerian studies, these PCR-positive samples were identified as *B. vogeli* and *B. rossi*, though *B. gibsoni*, also associated with *R. sanguineus* transmission in other parts of the world, was not detected confirming previous studies in Nigeria [51,52].

Prior reports of PCR positivity for *Erlichia* sp. (*E. canis*) from a variety of geographical locations range from 7 to 77% [20,21,22,46,52], with this study’s rate of 40.5% in the middle of prior estimates. Curiously, this study reported a lower seropositivity, 29.7% (lifetime exposure), than the PCR (current infection) rate in Nigerian dogs. However, this is probably explained by the large number of dogs under a year of age in this study cohort, such that they had yet to seroconvert.

*Anaplasma* sp. co-infection with *Erlichia* sp. was evident on both POC (serology) and PCR assays though at a much lower rate than *Erlichia* sp. Infection, with very few dogs identified to be positive for *Anaplasma* alone on serology (none on PCR). The POC test used will not distinguish between *A. phagocytophilum* and *A. platys* [25]. However, as the vector for *A. phagocytophilum*, *Ixodes ricinus*, is not present in Nigeria, the seropositivity in this study is likely due to *A. platys* (PCR products obtained in this study were too short for phylogenetic analysis and further species confirmation). As the *E. canis* and *A. platys* are transmitted by the same vector (*R. sanguineus*) [53], it is not surprising to see most *Anaplasma* infections occurring as co-infections with *Erlichia* sp. Co-infections may result in severe clinical disease [9,10] and confounding laboratory diagnosis [22,54,55]. This is a factor that should be considered during diagnosis and treatment of TBD in Nigerian dogs.

There was poor concordance between PCR and serology testing for these pathogens. This is not surprising as the POC test measures seropositivity (lifetime exposure), whereas the PCR assay identifies current infection. With a large number of young dogs assessed in this study, many will have been undergoing initial infection. This age structure is typical for the Nigerian dog population and, thus, PCR-based testing is likely more appropriate than serological tests for these pathogens in Nigerian dogs.

### 4.3. Low Prevalence of Dirofilaria sp. and the Absence of B. burgdorferi (s.l) Using POC and PCR Tests

A low prevalence of *D. immitis* was observed using both POC and PCR diagnostics. PCR screening detected no *Dirofilaria* sp. in Nigerian dogs. Commercial POC test kits cross-react with *D. repens* [26,27], and a higher prevalence was expected due to previous reports in Nigeria, (2.2–3.4%) [56]; however, some recent studies [51] have reported a complete absence of *Dirofilaria* sp. similar to this study. This is perhaps attributable to routine use of ivermectin promoted by veterinarians for routine deworming and the ongoing River Blindness (*Onchocerca volvulus*) programme in West Africa. Ivermectin is known to cause a “slow kill” effect, clearing microfilaraemia and resulting in negative PCR tests [57,58,59].

*B. burgdorferi* (s.l.) was not detected with either POC/PCR test results. *B. burgdorferi* (s.l.) has not been reported in Nigeria [53]. Some North African countries have reported seroprevalence to *B. burgdoferi* (s.l.) using IDEXX^®^ [60] and IFA [61] tests, but the absence of established tick vectors (*Ixodes* sp.) likely accounts for its absence in Nigeria [62,63].

### 4.4. Risk Factors Associated with Canine Tick Infestation and Tick-Borne Pathogens in Nigeria

The assessment of risk factors associated with canine tick-borne pathogen seropositivity demonstrated that young and adult dogs with tick infestation were more likely to be seropositive to TBPs than puppies and tick-free dogs, consistent with previous findings where age and presence of tick infestation were established to increase the risk of seropositivity to TBDs [64]. Additionally, male dogs exhibit a higher risk of tick infestation compared to females, a finding consistent with reports from Ayacucho, Peru, where males showed significantly greater prevalence of tick-borne infections [65]. This suggests that male dogs may engage in more outdoor activities, increasing exposure to ectoparasites. Male dogs have been associated with larger free-roaming populations [66]. Physiologically, elevated testosterone levels in males may weaken immune responses, predisposing them to higher parasitic loads [67].

The results of the risk factor assessment based on PCR prevalence (current infection) showed that non-indigenous (exotic) dog breeds were at significantly higher risk of testing positive to TBPs than indigenous dog breeds. Dogs were more likely to test positive to TBPs via PCR during the rainy season than the dry season of the year as expected. Although exotic dog breeds were over presented in this study, this study supports the commonly held perception by Nigerian veterinarians that indigenous dog breeds are less likely to show clinical disease or test positive for TBP than exotic breeds. Reporting bias may play a role in the increased numbers of exotic breeds testing positive, as these dogs are more valuable and more likely to be presented for veterinary care than indigenous dogs, though seroprevalence rates did not show breed specific risk factors. There may also be genetic factors responsible for inherited resistance to TBP infection in Nigerian dogs as previously reported for tropical breeds in other species such as cattle [68,69], though this remains to be demonstrated with genetics studies.

## 5. Limitation and Conclusions

While the findings of this study provide valuable insights, certain limitations should be considered when interpreting the results. The piroplasm 18S rRNA qPCR assay used amplifies a fragment of over 400 bp, which may reduce analytical specificity. Future studies could employ qPCR assays with shorter amplicons to improve specificity and efficiency, complemented by conventional PCR for generating longer sequences to enable accurate species identification. In addition, the use of blood samples preserved on FTA cards rather than fresh whole blood may have affected the detection of *Dirofilaria immitis*. PCR sensitivity depends on the presence of circulating microfilariae; therefore, negative results may reflect their absence rather than true infection [70,71]. Previous research has shown strong correlations between microscopic and PCR findings, including Knott’s technique, highlighting the value of integrated diagnostic approaches [72]. Incorporating these methods in future work would help overcome these limitations and provide a more accurate estimate of prevalence. Furthermore, precise georeferencing of cases was not possible because only approximate locations were available, and permissions to include specific geographical details were not sought. We acknowledge that spatial analysis could provide additional insights and will consider incorporating georeferencing in future studies where appropriate permissions and data are available.

This study highlights the predominance of tropical lineage *R. sanguineus* and *H. leachi* ticks in Nigerian dogs. The absence of *B. burgdorferi* suggests a minimal risk of Lyme disease, consistent with its primary vector absence in Nigeria. The prevalence of *Dirofilaria* sp. was unexpectedly low and may be due to extensive ivermectin use and availability in Nigeria; this warrants follow-up to confirm and should be interpreted with caution. The risk factors for tick-borne diseases in the serology and PCR-based models are consistent with older dogs having had more exposure to tick-borne pathogens over time and increased transmission during the rainy season. The results indicating that PCR positivity for these pathogens is higher in introduced breeds of dogs is intriguing and supports the commonly held belief that indigenous dogs are less likely to present with clinical disease due to TBP infection. The reasons for this may relate to the underlying husbandry of more valuable introduced exotic pets or in genetic resistance to TBP in Nigerian dogs and this warrants further exploration and must be interpreted with caution. PCR tests are preferred over POC serology in younger dogs, as they can detect the presence of the pathogen early in infection, even before antibodies are produced, ensuring more accurate and timely diagnosis.

Finally, given the zoonotic potential of vector-borne pathogens identified in dogs in this study, including *Dirofilaria*, integrated surveillance strategies are essential. A One Health approach that combines monitoring in domestic animals, wildlife, humans, and vectors will strengthen early detection and inform effective control measures [73].

## Figures and Tables

**Table 1:** Prevalence of TBPs in Nigerian dogs (*n* = 259) detected by POC and qPCR from blood samples; includes *Dirofilaria immitis* (mosquito-borne) detected only by POC.

**(A). Prevalence of Pathogens Detected by POC Test**			
**Pathogen**	**POC Positive (*n*)**	**Prevalence (%)**	**95% CI**
Single pathogen			
*Ehrlichia* sp.	77/259	29.7	24.3–35.5
*Anaplasma* sp.	28/259	10.8	6.9–14.7
*Dirofilaria* sp.	1/259	0.4	0.0–1.2
Total	106/259	40.9	
Mixed infection			
*Ehrlichia* sp. + *Anaplasma* sp.	20/259	7.7	
*Dirofilaria* + *Ehrlichia* sp.	1/259	0.4	
Total	21/259	8.1	
**(B). Prevalence TBPs Detected by qPCR**			
**Pathogen**	**qPCR-Positive (*n*)**	**Prevalence (%)**	**95% CI**
Single infection			
*Babesia* sp.	45/259	17.4	12.7–22.4
*Ehrlichia* canis	105/259	40.5	34.7–46.7
*Anaplasma platys*	2/259	0.8	0.00–0.02
Total	152/259	58.7	
Mixed infection			
*Babesia* sp. + *Ehrlichia* sp.	19/259	7.3	
*Ehrlichia* sp. + *Anaplasma* sp.	2/259	0.8	
Total	21	8.1	

**Table 2:** (A,B). Summary of logistic regression analysis of risk factors for canine tick-borne pathogens (A) via POC diagnostic kit and tick infestation (B) in Nigeria.

A	Variable	Adjusted OR	95% CI	*p* value
	Age			
	Young–adult	Reference		
	Puppy	0.3	0.15–0.64	0.002 *
	Ectoparasites (ticks)			
	Yes	Reference		
	No	1.7	0.99–3.03	0.054 *
B	Variable	Adjusted OR	95% CI	*p* value
	Sex			
	Male	Reference		
	Female	1.7	1.02–2.86	0.041 *

CI: confidence interval, OR: odd ratio, * *p* values statistically significant (*p* < 0.05).

**Table 3:** Summary of adjusted model on multivariate logistic regression analysis of risk factors associated with canine tick-borne pathogens detection using qPCR.

Variable	Adjusted OR	95% CI	*p* Value
Breed			
Exotic	Reference		
Indigenous	0.5	0.27–0.82	0.008 *
Season			
Rainy (April–October)	Reference		
Dry (November–March)	0.5	0.28–0.86	0.013 *

CI: confidence interval, OR: odd ratio, * *p* values statistically significant (*p* < 0.05).

**Table 4:** Percentage prevalence of pathogens detected, and Cohen’s Kappa index (95% CI) derived from POC and PCR-based tests on Nigerian canine blood samples.

Pathogen Species	POC Test: % (*n*)		PCR-Based Test: % (*n*)		*p* Value (KCI)
	Positive	Negative	Positive	PCR +ve from POC−ve	
*Ehrlichia canis*	29.7 (77)	70.3 (182)	40.5 (105)	28.6 (74)	0.9 (−0.04–0.06)
*Anaplasma*	10.8 (28)	89.2 (231)	0.8 (2)	0.4 (1)	0.7 (0.53–0.59)
*Dirofilaria*	0.4 (1)	99.6 (258)	0 (0)	0 (0)	Np
*Babesia*	Np	Np	17.4 (45)	Np	Np

Note: % (*n*): prevalence/negative results with sample number; K (CI): Cohen’s kappa index and 95% CI; PCR +ve from POC -ve: samples negative on POC but later PCR-positive; Np: omits calculations for single-assay detections.

## Data Availability

Nucleotide sequences obtained from this study were deposited in the NCBI GenBank database under accession numbers ON510072–ON510103 (*R. sanguineus*) and ON457742–ON457746 (*H. leachi*), (Supplementary File S1: Table S4).

## Supplementary Materials

The following supporting information can be downloaded at: https://www.mdpi.com/article/10.3390/pathogens14121271/s1. Supplementary File S1 consist of the following figures and tables: Figure S1: Agarose gel (1%) picture obtained following hard tick 16S RNA gene PCR; Figure S2: A flow chart showing sample collection, preservation, shipment, field and laboratory processing in Nigeria and the United Kingdom; Table S1: Showing confidence interval and sample size estimated by taking an arithmetic mean of published prevalence on CVBPs in Nigeria; Table S2: Summary of PCR protocols, primers, product size, and references for protocols used during PCR identification of hard ticks and screening of both tick and canine blood samples; Table S3: Summary of percentages and number of adult ticks collected from Nigerian dogs, based on morphological and 16S gene PCR, including number of adult ticks sequenced in this project; Table S4: Summary of variable factors and univariate analysis of potential predictors associated with canine tick infestation in Nigeria; Table S5: Summary of variable factors and univariate analysis of potential predictors associated with TBPs prevalence in Nigerian dogs; Table S6: Summary of variable factors and univariate analysis of potential predictors associated with CVBPs prevalence in Nigerian dogs based on qPCR screening test (*n* = 259); Table S7: GenBank accession number and list of hard tick species nucleotide sequences obtained from Nigerian dogs and domestic birds. Supplementary File S2: Dog sample collection form which include verbal consent sought from dog owners and a list of demographic data collected on all dogs assessed in this study.
