# Supplementary material for: Canine Ticks, Tick-Borne Pathogens and Associated Risk Factors in Nigeria
Source: Pathogens. 2025 Dec 11;14(12):1271. doi: 10.3390/pathogens14121271 (PMC12735562; doi:10.3390/pathogens14121271)
Supplement: Supplementary file 1 [file pathogens-14-01271-s001.zip › pathogens-3984787-supplementary.pdf]

Supplementary file S1

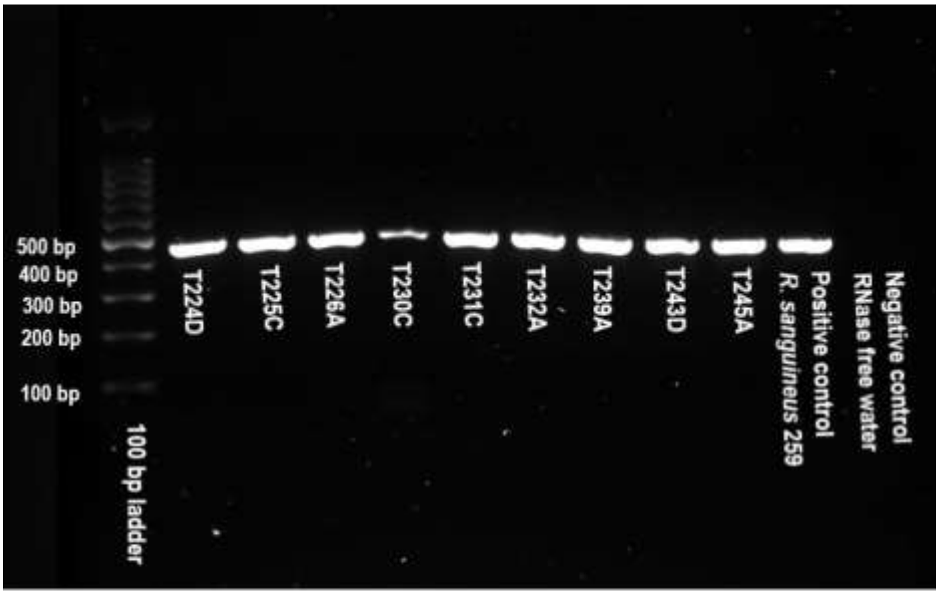

Figure S1: Agarose gel (1%) picture obtained following hard tick *16S RNA* gene PCR. From left to right (L-R), first lane: 100 bp DNA ladder, Lane labeled T224D to T245A (L-R), refers to hard tick samples collected from owned dogs. The last two lanes on the far right represent positive control (*R. sanguineus* DNA (courtesy of Dr Lesley Bell-Sakyi and team, The Tick Cell Biobank, University of Liverpool UK) and negative control (RNase free water), respectively.

Table S1: Summary of percentages and number of adult ticks collected from Nigerian dogs, based on morphological and 16S gene PCR, including number of adult ticks sequenced in this project.

Tick genera	Animal host, percentage (%) and number of adult ticks examined		Total percentage and number	
	Canine			Number of adult ticks sequenced
Rhipicephalus	95.5% (107/112)		95.5% (107/112)	35
Haemaphysalis	4.5% (5/112)		4.5% (5/112)	5
Total	100% (112/112)		100% (112/112)	40

Table S2. GenBank accession number and list of hard tick species nucleotide sequences obtained from Nigerian dogs and domestic birds.

Accession	Sequence ID	*Species*
ON510072	T8D	*R. sanguineus*
ON510073	T9A	*R. sanguineus*
ON510074	T10D	*R. sanguineus*
ON510075	T28A	*R. sanguineus*
ON510076	T29B	*R. sanguineus*
ON510077	T30C	*R. sanguineus*
ON510078	T31C	*R. sanguineus*
ON510079	T33B	*R. sanguineus*
ON510080	T35B	*R. sanguineus*
ON510081	T36A	*R. sanguineus*
ON510082	T37A	*R. sanguineus*
ON510083	T39D	*R. sanguineus*
ON510084	T52A	*R. sanguineus*
ON510085	T53A	*R. sanguineus*
ON510086	T55C	*R. sanguineus*
ON510087	T57B	*R. sanguineus*
ON510088	T89D	*R. sanguineus*
ON510089	T90D	*R. sanguineus*
ON510090	T103D	*R. sanguineus*
ON510091	T121C	*R. sanguineus*
ON510092	T140A	*R. sanguineus*
ON510093	T149A	*R. sanguineus*
ON510094	T150D	*R. sanguineus*
ON510095	T153D	*R. sanguineus*
ON510096	T160C	*R. sanguineus*
ON510097	T170C	*R. sanguineus*
ON510098	T172D	*R. sanguineus*
ON510099	T220C	*R. sanguineus*
ON510100	T221A	*R. sanguineus*
ON510101	T223D	*R. sanguineus*
ON510102	T231C	*R. sanguineus*
ON510103	243D	*R. sanguineus*

ON457742	T1D	*Haemaphysalis leachi*
ON457743	T3A	*Haemaphysalis leachi*
ON457744	T3B	*Haemaphysalis leachi*
ON457745	T4D	*Haemaphysalis leachi*
ON457746	T5A	*Haemaphysalis leachi*

Table S3: Showing confidence interval and sample size estimated by taking an arithmetic mean of published prevalence on CVBPs in Nigeria.

Sample Size for Frequency in a Population			
Population size (for finite population correction factor or fpc) (*N*):			1000000
Hypothesized % frequency of outcome factor in the population (*p*):			41.8%+/-5
Confidence limits as % of 100(absolute +/- %) (*d*):			5%
Design effect (for cluster surveys-*DEFF*):			1
Sample Size(*n*) for Various Confidence Levels			
Confidence	Level (%)	Sample Size	
95%		374	
80%		160	
90%		264	
97%		459	
99%		646	
99.9%		1053	
99.99%		1472	
Equation: Sample size n = [DEFF*Np(1-p)]/ [(d^2/Z$^2_{1-\alpha/2}$*(N-1)+p*(1-p)]			

Results from OpenEpi, Version 3, open-source calculator—SSPropor.

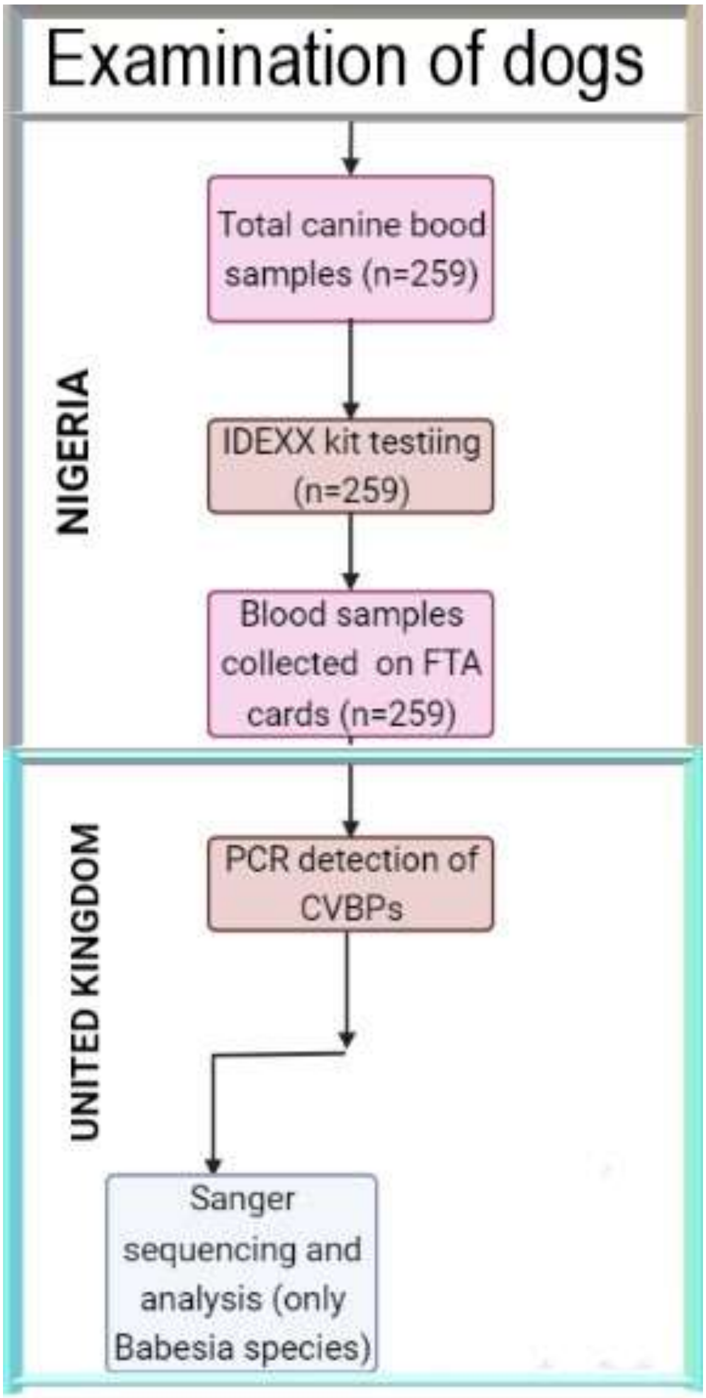

Figure S2: A flow chart showing sample collection, preservation, shipment, field and laboratory processing in Nigeria and the United Kingdom.

Table S4: Summary of PCR protocols, primers, product size, and references for protocols used during PCR identification of hard ticks and screening of both tick and canine blood samples.

PCR assay			Thermal cycling conditions (temperature, time, and cycles)					
	Primers	Size (bp)	Denaturation	Annealing	Extension	Final extension	cycles	References
	Conventional PCR assay							
Hard ticks	16S+1 (5'-CTGCTCAATGATTTTTTAAATTGCTGTGG-3')<br>16S-1 (5'-CCGGTCTGAACTCAGATCAAGT-3')	460	[a]94°C 3 min<br>[b]94°C 1 mi	54°C 30s	72°C 90s	72°C 5 min		(Black and Piesman, 1994)
Ehrlichia generic	GE2F (5′-GTTAGTGGCATACGGGTGAAT-3′)<br>EHRL3-IP2 (5′-TCATCTAATAGCGATAAATC-3′)	100	[a]94°C 3 min<br>[b]94°C 45s	50°C 30s	72°C 90s	72°C 90s	40	(Breitschwerdt et al., 1998)
	qPCR assay							
Ehrlichia	Ecp30-f (5’-TGGATACTACCATGGCGTTATTGG-3’)<br>Ecp30-r (GAGGAGCATCATTTAATACTACAGGAGTT-3’)	80	[a]98°C 3 min<br>[b]94°C 15 s	60°C 45s	[c]55-92°C @0.01s intervals	N/A	40	(Ionita et al., 2013)
Piroplasm	Piro-A (5'-AATACCCAATCCTGACACAGGG-3')<br>Piro-B (5'-TTAAATACGAATGCCCCCAAC-3')	408 – 430	“	“	“	N/A	“	(Armstrong et al., 1998, de Marco et al., 2017)
Borrelia	Bor16S3F (5′-AGCCTTTAAAGCTTCGCTTGTAG-3′)<br>Bor16S3R (5′-GCCTCCCGTAGGAGTCTGG-3′)	148	“	“	“	N/A	“	(Parola et al., 2011)
Dirofilaria	PanFilaF (5’-TGTGCTGCGCTACATCGATG-3’)<br>PanFilaR (5’-AAACCGCTCTGTCTCACGAC-3’).	90bp	“	“	“	N/A	“	(Kronefeld et al., 2014)
Note: [a]&[b] (initial denaturation and denaturation temperature and time); [c] (melting temperature); N/A (not applicable).								

Table S5: Summary of variable factors and univariate analysis of potential predictors associated with TBPs prevalence in Nigerian dogs (*n* = 259) using POC test results.

Variable	Level	Total tested (%)	POC test positive (%) for CVBPs	OR 95% CI	P-value
Age	Young and adult	196	74 (37.8%)	Reference	
	Puppy	63	11 (17.5%)	0.35, 0.17 ─ 0.71	0.004
Sex	Male	154	52 (33.8%)	Reference	
	Female	105	33 (31.4%)	0.90, 0.53 ─ 1.53	0.694
Breed	Exotic	179	56 (31.3%)	Reference	
	Indigenous	80	29 (36.3%)	1.25, 0.72 ─ 2.18	0.432
Body weight (kg)	≤20 kg	172	54 (31.4%)	Reference	
	≥21kg	87	31 (35.6%)	1.21, 0.70 ─ 2.09	0.493
Season of the year	Rainy (April - October.)	181	62 (34.3%)	Reference	
	Dry (November - March)	78	23 (29.5%)	0.80, 0.45 ─ 1.43	0.454
Presence of ectoparasites	Yes	153	57 (37.3%)	Reference	
	No	106	28 (26.4%)	1.65, 0.96 ─ 2.84	0.068

POC: Point-of-care test (IDEXX antigen test kit), CI: confidence interval, n: sample size, OR: odd ratio, ≤: less than or equal to, ≥ greater than or equal to, CVBPs: canine tick-borne diseases.

Table S6: Summary of variable factors and univariate analysis of potential predictors associated with canine tick infestation in Nigeria (*n* = 259).

**Variable**	**level**	**Total examined**	**Tick prevalence**	**OR 95% CI**	**P value**
Age	Young and adult	196	117 (59.7%)	Reference	0.720
	puppy	63	36 (57.1%)	0.90, 0.51 – 1.60	
Sex	Male	154	83 (53.9%)	Reference	0.041
	female	105	70 (66.7%)	1.71, 1.02 – 2.86	
Breed	Exotic	179	109 (60.9%)	Reference	0.373
	Indigenous	80	44 (55%)	0.79, 0.46 – 1.34	
Body weight (kg)	≤20 kg	172	106 (61.6%)	Reference	0.240
	≥21kg	87	47 (54.0%)	0.73, 0.43 – 1.23	
Season of the year	Rainy (April - October.)	181	109 (60.2%)	Reference	0.567
	Dry (November - March)	78	44 (56.4%)	0.86, 0.499 – 1.46	
Location	Benue	223	127 (57.0%)	Reference	0. .280
	Kaduna	14	9 (64.3%)	1.36, 0.44 – 4.19	
	Abuja	12	8 (66.7%)	1.51, 0.44 – 5.17	
	Ibadan	10	9 (90.0%)	6.80, 0.85 – 54.62	

CI: confidence interval, n: sample size, OR: odd ratio, ≤: less than or equal to, ≥ greater than or equal to.

Table S7: Summary of variable factors and univariate analysis of potential predictors associated with CVBPs prevalence in Nigerian dogs based on qPCR screening test (n = 259).

Variable	level	Total tested (%)	qPCR positive (%) for CVBPs	OR 95% CI	P value
Age	Young and adult	196	96 (49%)	Reference	
	puppy	63	34 (54%)	0.82, 0.46 ─ 1.45	0.491
Sex	Male	154	77 (50%)	Reference	
	female	105	53 (50.5%)	1.02, 0.62 ─ 1.67	0.940
Breed	Exotic	179	100 (55.9%)	Reference	
	Indigenous	80	30 (37.5%)	0.47, 0.28 ─ 0.81	0.007
Body weight (kg)	≤20 kg	172	84 (48.8%)	Reference	
	≥21kg	87	46 (52.9%)	1.18, 0.70 ─ 1.97	0.540
Season of the year	Rainy (April - October.)	181	100 (55.2%)	Reference	
	Dry (November - March)	78	30 (38.5%)	0.51, 0.29 ─ 0.87	0.014
Presence of ectoparasites	Yes	153	82 (53.6%)	Reference	
	No	106	48 (45.3%)	1.4, 0.85 ─ 2.29	0.189
Location	Benue	223	109 (48.9%)		
	Kaduna	14	5 (35.7%)		
	FCT	12	8 (66.7%)		
	Ibadan	10	8 (80%)	0.58, 0.19 ─ 1.79	0.134

CI: confidence interval, n: sample size, OR: odds ratio, ≤: less than or equal to, ≥ greater than or equal to, CVBPs: canine tick-borne diseases. The model to assess risk factors was built using information on all dogs that tested positive for all CVBPs (*Babesia* or *Ehrlichia* or both) rather than a singular pathogen.

Supplementary file S2

Sample collection form, Species: Canine

A. Identification and History. Date…………………………………………..

Sample serial no: ……….Location/GPS codes: ……………………………………………………………

History of present illness: …………………………………………………………………………………………….

…….

………..

Previous treatment history (including flea/ticks/worm products used): …………………………..

…….

Source of sample: Clinic ☐ Veterinarian ☐

Others (specify) ………………………………………………………………………………………….

ARV Vaccination: Yes ☐ No ☐ Please specify the ARV brand used: ……………………………

DHLPP vaccination Yes ☐ No ☐ Please specify the ARV brand used: ……………………………

Sex: Female ☐ Male ☐ spayed (F) ☐ Castrate (M) ☐

Age: Puppies (<6 months) ☐ Young (<18 months) ☐ Adult (>18 months) ☐

Breed: Native ☐ Cross ☐ Alsatian ☐ Rottweiler ☐ Mongrel ☐

Others (specify)………………………………………………….. (Upload photo via facebook messenger)

Body condition score (1-5 scale attached)………………Body Weight…………….............kg

Dog's movement: Stray ☐ Non-stray ☐ Not known ☐

Level of Tick infestation: mild (1-5ticks) ☐ moderate (5-10-ticks) ☐ Heavy (>10 ticks)

Total number of ticks collected: …………………………………………..

Tick species identified morphologically……………………………………………… (Pls, send us photos)

Tentative Diagnosis: ………………………………………………………………………………..

Blood parasite identified via Quick diff stain/microscopy: ……………………………………………

(Please, send your pictures via svxtta@nottingham.ac.uk)

Number of parasites per field: ……………………………………………………………….…………………

B. 4Dx SNAP test results:

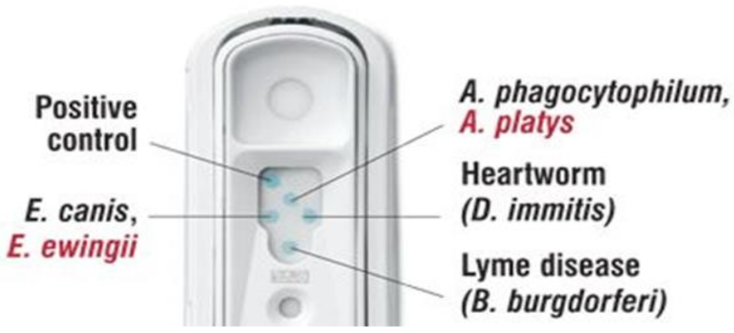

Heartworm ☐ Lyme disease ☐ *Ehrlichia* ☐ *Anaplasma* ☐

No of ticks squashed on FTA card ☐ Blood smeared on FTA cards ☐
